# Microstructure and Properties of Different Modulus Sections in JG4246A Alloy Characteristic Simulation Castings

**DOI:** 10.3390/ma19050915

**Published:** 2026-02-27

**Authors:** Hai-Tao Jiang, Lei Jin, Gao-Yang Jing, Peng Li, Bing-Zheng Fan, Yi-Peng Li, Lan-Bo Ma, Ao-Qi Li, Tian-Yv Liu, Xun Sun, Yang Guan

**Affiliations:** 1 China Academy of Machinery Shenyang Research Institute of Foundry Co., Ltd., Shenyang 110022, China; jht741128@163.com (H.-T.J.); jgysteel@163.com (G.-Y.J.); 13756972779@163.com (P.L.); fbzopm@163.com (B.-Z.F.); liyipeng945536084@gmail.com (Y.-P.L.); mlb1285417023@163.com (L.-B.M.); 15531064548@163.com (A.-Q.L.); liutianyusrif@163.com (T.-Y.L.); sx@foundrynations.com (X.S.); gy79060977@163.com (Y.G.); 2 National Key Laboratory of Advanced Casting Technologies, Shenyang 110022, China

**Keywords:** JG4246A alloy, different modula, microstructure, mechanical property, crack

## Abstract

This study takes the commercial JG4246A cast Ni_3_Al-based superalloy as the research object, under the conditions of preheating the mold shell at 1020 °C and a pouring temperature of 1520 °C, characteristic simulation castings were poured. The microstructure and room temperature mechanical properties of different modulus sections of the castings were systematically investigated. It was found that, except for the edge towards the middle section of the larger modulus, the cooling rates at the edge were greater than those at the middle sections. The cooling rate was the fastest at the upper-right corner section (referring to the castings position during pouring, the same below), and the grain is the finest (approximately 0.46 mm), with the highest strength (tensile strength approximately 698 MPa, yield strength approximately 581 MPa), while the cooling rate at the lower-middle section was the slowest, and the grain was the largest (approximately 1.55 mm), with the lowest strength (tensile strength approximately 612.5 MPa, yield strength approximately t 524.5 MPa); the difference in grain size between the two is nearly 237%. The MC carbides at the lower-edge middle section have the smallest size (approximately 3.0 μm) and the elongation rate in this area is the highest (approximately 8.7%), while the MC carbides at the lower-middle section have the largest size (approximately 5.8 μm) and the elongation rate in this area is the lowest (approximately 4.9%); the size difference in the MC carbides between two is nearly 94%. This study clarifies the quantitative correlation between cooling rate, microstructure and properties, providing clear guidelines for optimizing the casting process of high-temperature alloys and subsequent studies on the uniformity of microstructure.

## 1. Introduction

The density of the cast Ni_3_Al-based alloy JG4246A (original grade MX246A) is only 7.83 g∙cm^−3^, which can significantly reduce the structural weight of the engine and bring direct economic benefits [1,2,3]. This alloy exhibits excellent high-temperature strength and oxidation resistance within the range of 1000 to 1200 °C, allowing for long-term service without the need for thermal barrier coatings [4]. When the service temperature exceeds 1100 °C, its comprehensive performance still ranks among the top of the existing equiaxed-grained cast superalloys [5], and it has been used in batches for key hot-end castings such as regulating plates and sealing plates in the nozzles of aero engines [6].

A number of studies have confirmed that the microstructure–property correlation of superalloy components has a significant positional dependence. Kong et al. [7] conducted a study on the radial forged GH4169 alloy, which indicated that after standard heat treatment, the grain size in the edge zone was the smallest and the density of the precipitated phase at the grain boundaries was the highest. Under creep conditions of 650 °C/725 MPa, the life of this region is the shortest, while 1/2R is comparable to that of the core, verifying the dominant role of “fine grain weakening” in high-temperature creep. Li et al. [8] systematically characterized the GH4742 wheel forging and found that the secondary/tertiary γ′ precipitates on the end face were in a fine elliptical shape, while the upper and middle parts evolved into large petal-like shapes. The hardness shows a gradient of “high at both ends and low in the middle” along the axial direction, and monotonically decreases from the rim to the hub, directly determining the load-bearing margin of each part of the disk. Zhang et al. [9] used pressure-regulating casting (APC) to form large thin-walled nickel-based castings. They further revealed that due to the fast cooling rate in the thin-walled zone, both the grains and the γ′ phases were significantly refined, and the yield strength was increased by approximately 12% compared to the thick-walled zone of the flange. This indicates that the castings with different wall thicknesses can be locally strengthened and toughened by regulating the solidification paths. Liu et al. [10] conducted a systematic study on different regions of the K439B high-temperature alloy combustion chamber shell casting. They found that the maximum difference in creep rupture life (CRL) among different thin and thick regions of the casting was as high as 60%. However, the differences in the solidification sequence and the ability to feed at different shapes and positions also affected CRL. The main reason for the excellent CRL was that different wall thickness and shape regions could refine the dendritic structure at a faster cooling rate, and resulting in a small size and high volume fraction of γ′. The above studies consistently point out that even after the same heat treatment, different positions of the component will still form scale-morphology gradients of different sizes due to different solidification histories, deformation amounts and cooling rates, which in turn leads to the localized distribution of mechanical properties. For low-density Ni_3_Al-based alloys like JG4246A, the non-uniform thermal and mechanical loads that the castings are subjected to during service couple with their own complex structures and variable cross-section characteristics, making the “positional correlation performance” issue even more prominent. Therefore, systematic acquisition of microstructural characteristics and mechanical response data across different modulus regions is essential for establishing accurate process design and reliability assessment models for castings.

Most of the current studies on the JG4246A casting alloy are based on single-cast test bars, and their mechanical properties and microstructure data do not reflect the actual conditions of the casting bodies in complex structures. To fill this research gap, this study takes cast Ni_3_Al-based alloy parts with typical characteristics as the object, systematically segments different modulus regions, characterizes their microstructures and tests mechanical properties, and analyzes the grain sizes in combination with ProCAST 14.5 simulations of the temperature field distributions at characteristic positions, aiming to provide a theoretical and experimental data bases for the process optimization and reliability evaluation of castings.

## 2. Materials and Methods

**Figure 1:**
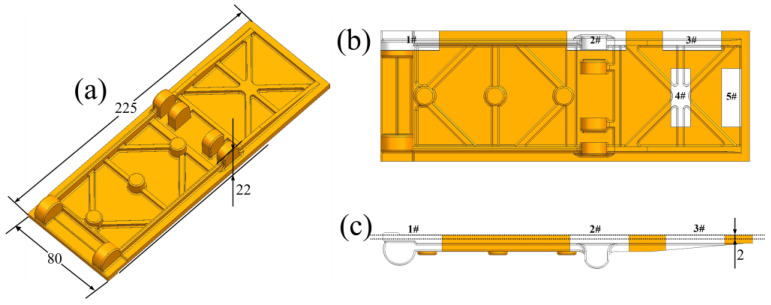
Isometric view of three-dimensional model and different feature areas/mm: (**a**) isometric view of three-dimensional model; (**b**) different feature areas; (**c**) side view and cutting line of mechanical specimen.

**Figure 2:**
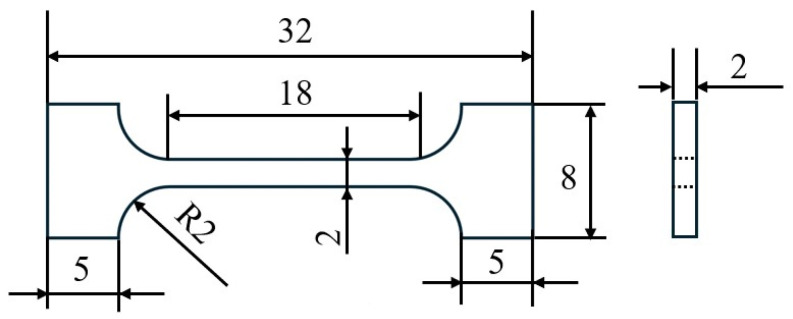
Mechanical specimen relevant dimensions/mm.

This test adopted the commercial JG4246A master alloy produced by a domestic factory, whose composition is shown in Table 1. The master alloy was melted in a 25 kg vacuum induction melting furnace and the characteristic simulation castings were poured. The three-dimensional model of the casting is shown in Figure 1a. The pouring temperature was 1520 °C, and the preheating temperature of the alumina ceramic shell was 1020 °C. After the casting was formed, the residual ceramic shell on the surface of the casting was removed through cutting, sandblasting and grinding. Then, metallographic and mechanical samples were cut from five characteristic parts by electrical discharge wire cutting. The positions of characteristic parts are shown in Figure 1b, and the corresponding maximum wall thicknesses and moduli are shown in Table 2. After the metallographic specimens were successively ground with 180#, 400# and 1200# SiC sandpapers and polished, they were etched with 2.5 mL H_2_SO_4_ + 10 g CuSO_4_ + 40 mL H_2_O + 50 mL HCl etching solution. The microstructure was observed using a stereomicroscope (MZ10F) and an optical microscope (Axio Vert. A1). After electrolytic corrosion solution with 84 mL of H_3_PO_4_ + 68 mL of H_2_SO_4_ + 48 mL of H_2_O etching for 3 to 5 s at a voltage of 5 volts, the γ′ phase observation was conducted using EVOMA25 (The EVOMA25 equipment is manufactured by Carl Zeiss AG in Germany, with the company’s corporate headquarters situated in Jena.) type SEM. The metallographic testing standards comply with Chinese Standard [11]. Five metallographic photos were selected for each feature section, and the grain size was calculated by the area method. A tensile test at room temperature (in accordance with the Chinese Standard [12], which is similar to the International Standard [13].) was conducted using a tensile machine of model ETM105D (The ETM105D equipment is manufactured by Shenzhen Wance Testing Machine Co., Ltd., which is headquartered in Shenzhen City, China). Two mechanical property specimens were selected for each characteristic part and at a strain rate of 3 × 10^−3^ s^−1^. The fracture surface and the area near the fracture surface were analyzed, respectively, by SEM and OM. The dimensions of the mechanical specimens are shown in Figure 2. The cutting line of the mechanical specimens is shown in Figure 1c. The cooling rates of different parts were simulated using ProCAST software.

**Figure 3:**
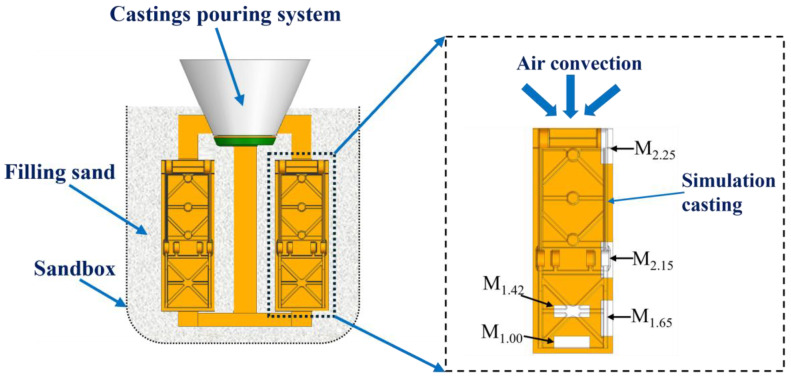
Schematic diagram of the placement of the simulation casting.

The modulus refers to the ratio of the volume V of a certain part of the casting to the effective heat dissipation area A, that is, M = V/A [14]. Under certain conditions, different modulus sections can be used to reflect the differences in the solidification sequence of various regions of the casting. Generally, the larger the modulus, the longer the solidification time. During the mold filling and solidification processes, the different modulus parts of the characteristic simulation casting have different spatial position distributions, and Figure 3 is a schematic diagram of the placement of simulation casting (Different parts are represented by M_x_, and x represents the modulus of the corresponding position).

## 3. Results

### 3.1. Grain Morphology and Size

**Figure 4:**
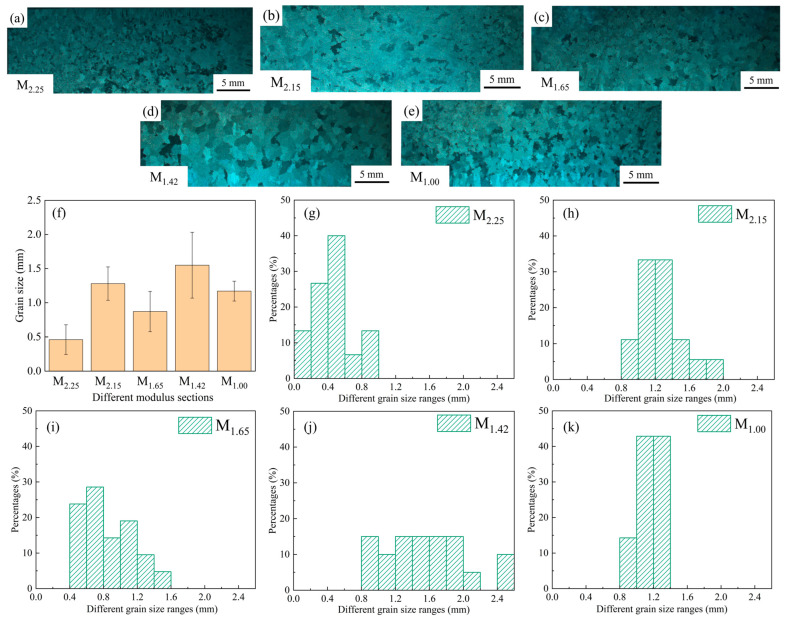
Grain morphologies of (**a**) M_2.25_; (**b**) M_2.15_; (**c**) M_1.65_; (**d**) M_1.42_; (**e**) M_1.00_ by stereomicroscope, sizes and percentages of grains of different sizes in (**f**–**k**) different modulus sections.

Stereomicroscopic analysis was conducted to study the grain morphology and size differences in each modulus part, as shown in Figure 4a–e. The results show that each modulus part presents an equiaxed crystal structure morphology, but there are significant differences in grain size. The grains in the M_2.25_ (Figure 4a) area are the finest, with an average size of approximately 0.46 mm. The grains in the M_1.42_ (Figure 4d) area are the coarsest, with an average size of approximately 1.55 mm. The grain sizes of the other parts fall between the two. Among them, the grain size of the M_1.65_ (Figure 4c) part is approximately 0.87 mm, that of the M_1.00_ (Figure 4e) part is approximately 1.17 mm, and that of the M_2.15_ (Figure 4b) part is approximately 1.28 mm. Overall, the grain size difference between the M_2.25_ and M_1.42_ sections is the most prominent. The average size of the former is only 30% of that of the latter, reflecting significant differences in solidification paths and cooling rates among different characteristic sections, which leads to significant unevenness in grain growth degree and refinement effect [15]. In addition, the grain distribution at the M_1.00_ part is relatively uniform, with a grain size fluctuation value of ±0.1 mm, while that at the M_1.42_ part is ±0.5 mm. This indicates that the different solidification conditions caused by the geometric size differences at different parts also have a certain impact on the uniformity of grain size. The grain size and fluctuation situation are shown in Figure 4f–k.

### 3.2. Dendrite Morphology, Eutectic Structure and MC Carbides Size

**Figure 5:**
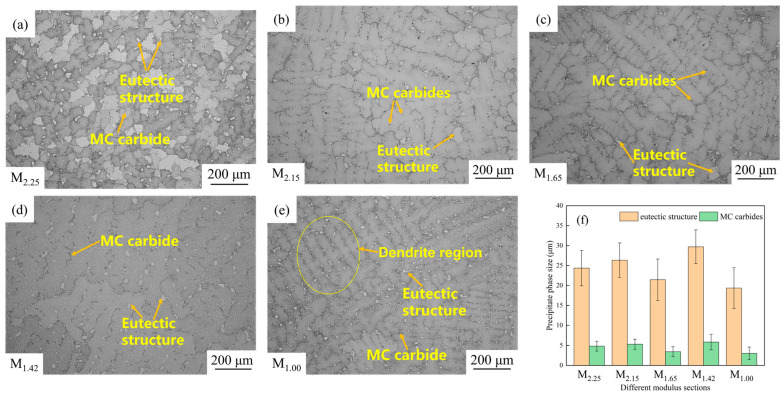
Microstructures of (**a**) M_2.25_; (**b**) M_2.15_; (**c**) M_1.65_; (**d**) M_1.42_; (**e**) M_1.00_ by OM and the sizes of (**f**) eutectic structure and MC carbides.

The dendrite structures and the metallographic microstructures of the precipitated phases at different modulus parts are shown in Figure 5a–e. Clear dendrite structures can be observed in each part of M_2.15_ (Figure 5b), M_1.65_ (Figure 5c), M_1.42_ (Figure 5d), and M_1.00_ (Figure 5e). However, the dendrite features of M_2.25_ are not obvious, and no typical dendritic morphology is seen, indicating that the dendrite growth during its solidification process is inhibited. Therefore, in each modulus part, there are generally many massive MC carbides and a eutectic structure between dendrites and near grain boundaries, and the overall distribution is relatively dense and uniform. The characteristic dimensions of the eutectic structure and MC carbides are shown in Figure 5f. The M_2.25_ site is approximately 24 μm and 4.8 μm, the M_2.15_ site is approximately 26 μm and 5.3 μm, the M_1.65_ site is approximately 21 μm and 3.4 μm, the M_1.42_ site is the largest, approximately 30 μm and 5.8 μm, while the M_1.00_ site is the smallest, approximately 19 μm and 3.0 μm. A comprehensive comparison shows that the size of MC carbides increases with the increase in eutectic structure. At the M_1.42_ site, not only is the dendrite structure clear, but also the size of the eutectic structure and MC carbides is the largest. On the contrary, the eutectic structure and MC carbides at the M_1.00_ site are the finest. It can be seen from this that different modulus parts show significant differences in dendrite morphology and eutectic structure, as well as MC carbides size, reflecting the differences in local solidification, cooling rate and the degree of composition segregation in each part. This will have an important impact on the subsequent mechanical properties [16,17].

### 3.3. γ′ Size and Morphology

**Figure 6:**
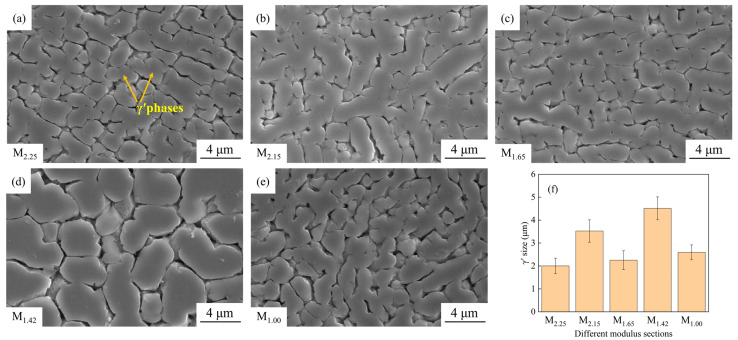
The γ′ phase morphologies of (**a**) M_2.25_; (**b**) M_2.15_; (**c**) M_1.65_; (**d**) M_1.42_; (**e**) M_1.00_ by SEM and sizes at (**f**) different modulus sections.

As shown in Figure 6, there are certain differences in the SEM morphologies and sizes of the γ′ phases in different modulus parts. Overall, the γ′ phases in each part mainly precipitate in L-shaped and blocky forms, with a relatively dense distribution. In terms of size, the γ′ phase at the M_2.25_ site (Figure 6a) is relatively fine, approximately 2.00 μm, while the γ′ phase at the M_1.42_ site (Figure 6d) is significantly larger, approximately 4.51 μm. The γ′ phase dimensions of the other modulus parts fall between the two. The M_2.15_ part (Figure 6b) is approximately 3.52 μm, the M_1.65_ part (Figure 6c) is approximately 2.25 μm, and the M_1.00_ part (Figure 6e) is approximately 2.59 μm. Based on the aforementioned analysis of grain and dendrite structures, it can be inferred that the differences in local cooling conditions and solidification precipitation kinetics at different modulus sites lead to different degrees of γ′ phase growth [18,19,20]. Especially, the γ′ phase size contrast between the M_2.25_ and M_1.42_ parts is quite prominent, which will have a significant impact on the mechanical properties of the alloy [21,22].

### 3.4. Mechanical Properties

**Figure 7:**
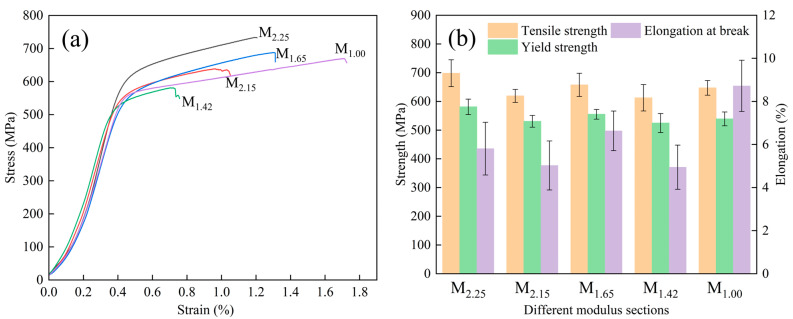
Engineering stress–strain curves (**a**) and mechanical properties (**b**).

Figure 7 shows the engineering stress–strain curves and mechanical properties of different modulus parts under tensile stress at room temperature. As shown in Figure 7b, the tensile strength and yield strength at the M_2.25_ section are both at their maximum, reaching approximately 698 MPa and 581 MPa, respectively. The tensile strengths of the M_1.65_ and M_1.00_ sections are close, approximately 658 MPa and 647 MPa, respectively, which are at a medium level. The tensile strengths of the M_2.15_ and M_1.42_ sections are relatively low, approximately 619 MPa and 613 MPa, respectively. Except for the M_2.25_ section, the yield strength of the M_1.65_ section is relatively high, approximately 555 MPa. The rest of the sections are not much different, with yield strengths of approximately 531 MPa, 525 MPa and 539 MPa, respectively. Overall, the strength of the M_2.25_ section is the highest, while that of the M_1.42_ section is the lowest. In terms of tensile strength, the M_2.25_ section is 13.96% higher than the M_1.42_ section, and in terms of yield strength, the M_2.25_ section is 10.77% greater than the M_1.42_ section. The tensile strength and yield strength both follow the order: M_2.25_ > M_1.65_ > M_1.00_ > M_2.15_ > M_1.42_, which corresponds to the line graph on the left side of Figure 7a. In Figure 7b, the elongation rate of the M_1.00_ part is the highest (approximately 8.7%), and that of the M_1.42_ part is the lowest (approximately 4.9%). The elongation of the M_2.15_ part (approximately 5.0%) is not much different from that of the M_1.42_ part, while the M_2.25_ (approximately 5.8%) and M_1.65_ (approximately 6.6%) parts are at a medium level.

## 4. Discussion

### 4.1. Numerical Simulation Analysis

As shown in Figure 4f, the relationship of grain sizes in different modulus parts is: M_1.42_ > M_2.15_ > M_1.00_ > M_1.65_ > M_2.25_. However, under the same conditions, the larger the modulus of the characteristic part, the slower the local cooling rate, and the grain size should be larger [15,23]. Therefore, from the perspective of modulus alone, the grain size should satisfy: M_2.25_ > M_2.15_ > M_1.65_ > M_1.42_ > M_1.00_. It can be seen that the actual measured grain size distribution has significant differences from the theoretical prediction obtained solely based on the modulus, and it is necessary to further analyze the combined effects of various influencing factors.

**Figure 8:**
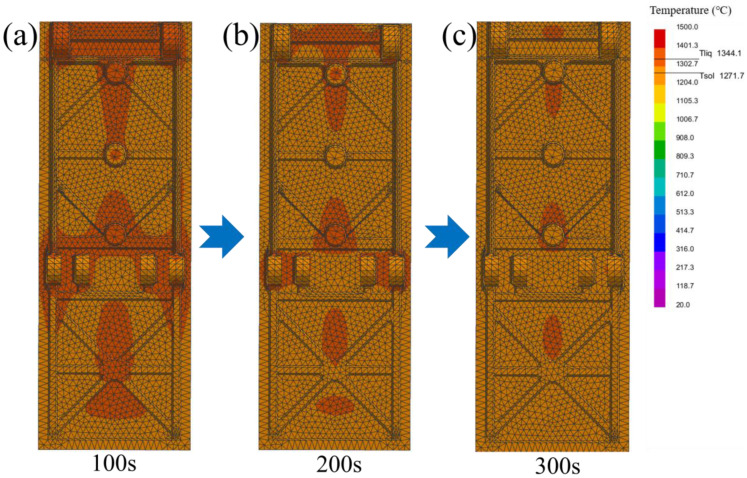
Temperature field changes in the simulation casting at (**a**) 100 s; (**b**) 200 s; (**c**) 300 s.

Figure 8 shows the numerical simulation results obtained by using the ProCAST software. From left to right of the figure, it shows the changes in the simulation casting arranged in chronological order with temperature fields. During the solidification process, the edges of the simulation casting come into direct contact with the mold shell, causing the edge temperature to drop rapidly and initiating solidification first. As heat is transferred from the interior of the simulation casting to the already solidified edge, the solidification process gradually proceeds inward. As shown in Figure 3 and Figure 8a, M_2.25_ is located at the very top of the casting, with the closest distance to the air above, and the convective heat transfer is the most intense [24]. At the same time, this position is at the edge of the casting, which is conducive to priority heat dissipation. The hot spot around it is less than that of M_1.65_, and the cooling rate is the fastest. The M_1.65_ position has a relatively small modulus and is located at the edge of the casting, with a cooling rate second only to M_2.25_. The M_1.00_ area has the smallest modulus and cools relatively quickly. However, there are still some hot spots nearby, and the cooling rate is lower than that of M_1.65_. M_2.15_ and M_1.42_ cool more slowly. At 200 s (Figure 8b), the hot spot near M_1.00_ has significantly decreased, while some areas of M_2.15_ are still solidifying and cooling. Therefore, the cooling rate of M_1.00_ is better than that of M_2.15_. Subsequently, at 300 s (Figure 8c), it can be seen that the rest of the parts are gradually solidifying and cooling down. However, there is still a small amount of hot spot near M_1.42_, so the cooling rate is the slowest.

According to the classical nucleation theory, the critical nucleation size, critical nucleation work, and nucleation rate are expressed as follows [15]:


(1) $$r^{*}=\frac{2\sigma T_{m}}{∆H_{V}∆T}$$

(2) $${∆G}^{*}=\frac{16\pi \sigma^{3}{T_{m}}^{2}}{3∆{H_{V}}^{2}{∆T}^{\;2}}\;\;$$

(3) $$I=K\cdot e^{-∆G^{*}/kT}\cdot e^{-Q/kT}\;\;$$

Here, r* is the critical nucleation radius, with the unit (m); *σ* is the liquid–solid interfacial energy per unit area, with the unit (J·m^−2^); *T*_m_ is the melting point of a metal, with the unit (K); Δ*H*_V_ is the enthalpy of fusion per unit volume of the melt, with the unit (J·m^−3^); Δ*T* is the degree of undercooling, with the unit (K); ΔG* is the critical nucleation work, with the unit (J·m^−3^); K is a proportionality constant, with the unit (s^−1^·m^−3^); *I* is the nucleation rate, with the unit (s^−1^·m^−3^); k is the Boltzmann constant; *T* is the nucleation temperature, with the unit of (K); *Q* is the diffusion activation energy of a metal atom through the solid–liquid interface, with the unit (J·m^−3^). During the solidification process of molten metal, as the cooling rate continuously increases, on the one hand, the degree of subcooling keeps increasing, which reduces the critical nucleation radius and nucleation work, significantly enhancing the nucleation rate and increasing the number of crystal nuclei formed per unit volume. On the other hand, rapid cooling shortens the solidification time and inhibits the grain growth process. The combined effect of the two leads to a continuous reduction in grain size.

**Figure 9:**
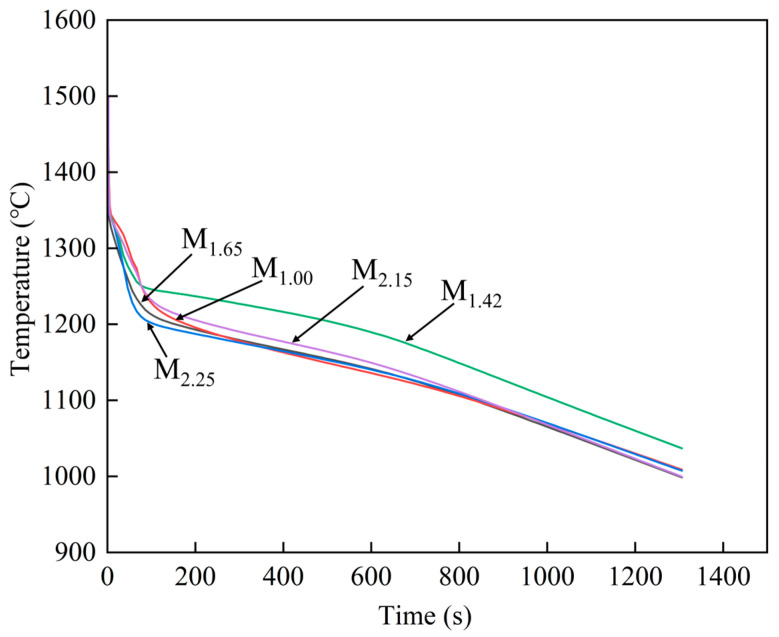
Temperature variation curves of feature points at different locations over time.

Based on the ProCAST simulation results, the temperature variation curves of characteristic points in different modulus parts with time can be obtained, as shown in Figure 9. By comparing the cooling curves at different positions, the differences between the above-mentioned temperature fields and the cooling rates can be further quantitatively analyzed. This quantitatively corroborated the grain size trend in Figure 4f and explained the deviation from predictions based solely on modulus.

### 4.2. The Correlation Between Microstructure and Mechanical Properties

According to the Hall-Petch formula [25], it can be known that the yield strength of the alloy will decrease as the grain size increases, that is,


(4) $$\sigma =\sigma_{i}+k_{y}d^{\;-\frac{1}{2}}$$

Here, *σ* represents the yield strength of the metallic material; *σ*_i_ represents the frictional resistance of grains to dislocation movement; *k*_y_ represents the degree of stress concentration caused by dislocations accumulating at grain boundaries, which is related to the number of effective slip systems; *d* represents the grain diameter. Figure 4f shows the comparison relationship of grain sizes in different modulus parts: M_2.25_ < M_1.65_ < M_1.00_ < M_2.15_ < M_1.42_, which is negatively correlated with the corresponding yield strength, conforming to the variation law in the Hall-Petch formula.

The finer the γ′ phase size in nickel-based alloys, the stronger the hindrance to dislocations, and the higher the yield strength of the material [26]. The relatively fine (2.00 μm) and compact-distributed γ′ phases at the M_2.25_ site are conducive to providing a better strengthening effect [27], while the relatively coarse (4.51 μm) γ′ phases at the M_1.42_ site may reduce the dislocation pinning ability, thereby weakening its strengthening effect [28]. As shown in Figure 6f, the yield strengths of different modulus regions increase as the γ′ phase sizes decrease. Figure 6f and Figure 4f show γ′ phase size is positively correlated with the grain size [29]; as the average grain size decreases, the γ′ phase size also decreases accordingly, which is in line with the variation law in relevant literature [30]. As can be seen from Figure 7b, the variation laws of the tensile strength and yield strength of the alloy are consistent. Therefore, the grain size and γ′ phase size jointly affect the tensile and yield strengths of the alloy materials. The smaller the grain size and γ′ phase size, the higher the strength of the corresponding material.

**Figure 10:**
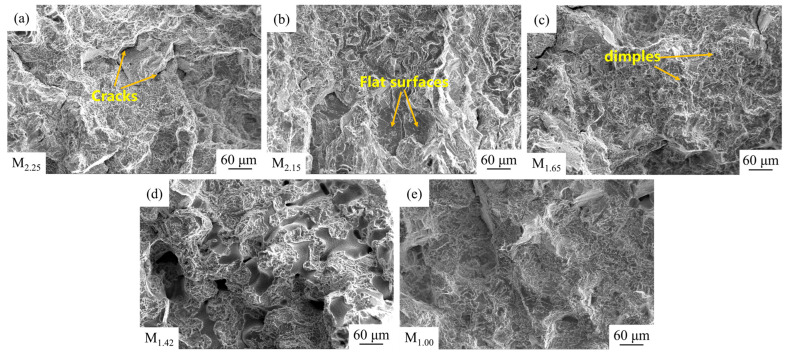
SEM images of tensile fracture surfaces: (**a**): M_2.25_; (**b**): M_2.15_; (**c**): M_1.65_; (**d**): M_1.42_; (**e**): M_1.00_.

Figure 10 shows the fracture microstructure morphologies. There are a large number of flat surfaces at each modulus part in the figure, and some of them converge towards the crack propagation direction, which has typical brittle characteristics. There are also some dimple-like structures, indicating the presence of some plasticity. Overall, it is a mixed fracture, but the brittle fracture feature is more obvious, which is related to the dense distribution of a large amount of γ′ phases in the microstructures. In superalloys, strength and plasticity are in contradiction. While a large amount of γ′ phases are formed, they are also accompanied by the precipitation of brittle phases such as eutectic structure and carbides, which hinders the deformation capacity of the alloy and reduces its plasticity [31]. The sizes of the eutectic structure and MC carbides have a significant impact on the plasticity of the alloy [16]. M_1.00_ has more dimples and certain plasticity, while M_1.42_ has a large number of flat surfaces and almost no dimple structure can be seen. It can be known from Figure 5f and Figure 7b that the sizes of eutectic structure and MC carbides at the M_1.00_ site are the smallest (about 19 μm and 3.0 μm, respectively), with the least fracture tendency and the highest elongation. The sizes of the eutectic structure and MC carbides at the M_1.42_ site are the largest (approximately 30 μm and 5.8 μm, respectively), and the elongation is the smallest. As shown in Figure 7b, the elongation at M_2.15_ is slightly higher than that at M_1.42_, and more flat surfaces can be seen near the fracture surface. In Figure 5f, it can be seen that the sizes of the eutectic structure and MC carbides are also relatively large (about 26 μm and 5.3 μm, respectively), which leads to poor alloy plasticity. The M_2.25_ and M_1.65_ sites are at a medium level, but M_1.65_ has more dimples than M_2.25_ and a lower degree of brittle fracture. As shown in Figure 5f and Figure 7b, the sizes of the eutectic structure and MC carbides (about 21 μm and 3.4 μm, respectively) of M_1.65_ are smaller than those of M_2.25_ (about 24 μm and 4.8 μm, respectively), leading to a smaller tendency to fracture, and the elongation is greater than that of the M_2.25_ part.

### 4.3. Microstructure Analysis near the Fracture Surface

**Figure 11:**
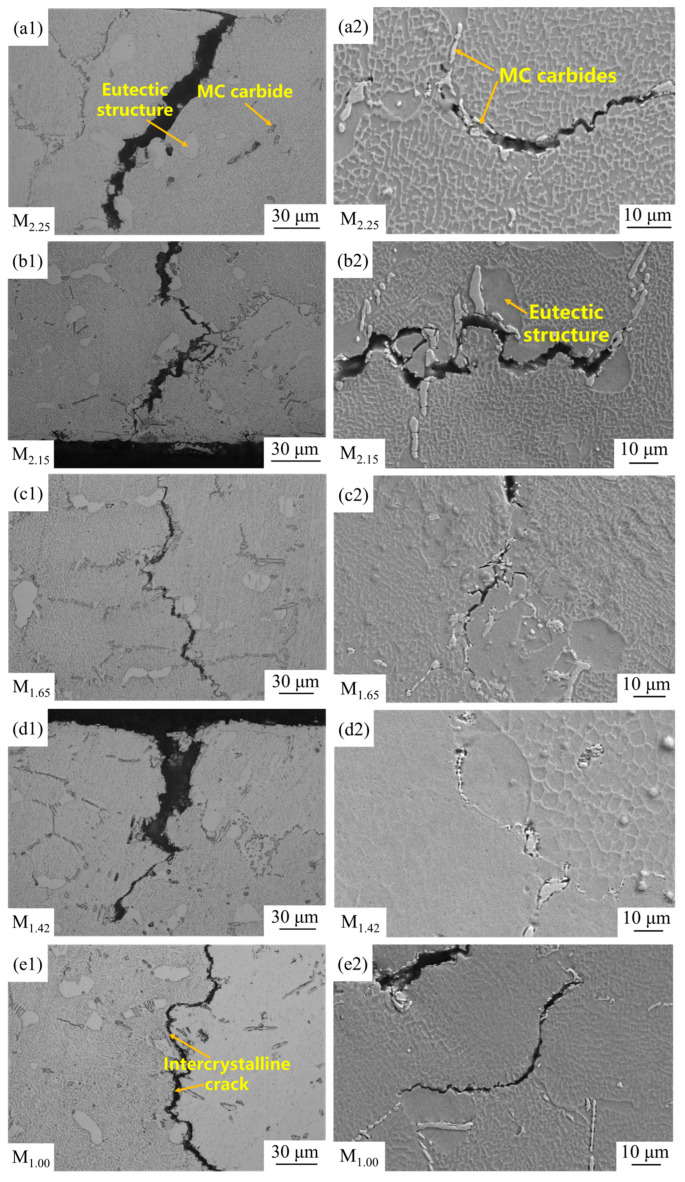
Microstructures near the fracture surfaces by SEM: (**a1**,**a2**) M_2.25_; (**b1**,**b2**) M_2.15_; (**c1**,**c2**) M_1.65_; (**d1**,**d2**) M_1.42_; (**e1**,**e2**) M_1.00_.

The left side of Figure 11a1–e1 shows the low-magnification structures near the stretched zones, and the right side of Figure 11a2–e2 shows the microstructures at high magnification. In the low-magnification structures, the white blocky areas near the cracks are eutectic structures, and the black dot areas are MC carbides. In the high-magnification structures, the white dot and strip areas are MC carbides, and the large blocky dark gray areas are the eutectic structure. In Figure 11a1–e2, there is a large amount of eutectic structure and carbides distributed in the dendrite regions and grain boundaries. This is because the atomic structure arrangement between dendrites and at grain boundaries is loose, with higher energy, which is more conducive to the formation of new phases. Meanwhile, the diffusion rates of alloying elements at grain boundaries and in dendrites are faster than that within grains. C atoms and other metal elements rapidly migrate and aggregate, quickly reaching the required concentration for eutectic structure and carbides and precipitating [32]. A large number of eutectic structures and carbides belong to the brittle phases, which are prone to inducing crack formation. As can be seen from Figure 11a1–e2, there is no significant difference in crack distribution at different modulus locations, mainly initiating cracks in the eutectic structure and MC carbides near dendrites and grain boundaries [33].

Under high-temperature tensile conditions, due to the enhanced atomic activity in the high-temperature environment, the atomic migration rate at the grain boundaries accelerates, leading to grain boundary diffusion and sliding. When the temperature exceeds the isothermal temperature, the strength of the grain boundaries decreases much more than that within the grains, resulting in grain boundary weakening [34]. Therefore, superalloys usually exhibit intergranular fracture in high-temperature environments, while during room-temperature tensile, the strength at the grain boundaries is greater than that within the grains, and it is usually transgranular fracture. However, although intergranular fracture is more typical at elevated temperatures due to grain boundary diffusion and sliding, cracking can also be observed near grain boundaries in the present room-temperature tests (Figure 11a1–e2). This is mainly because the interfaces between eutectic structure/MC carbides and the matrix at/near grain boundaries act as preferential weak sites. Dislocation accumulation around these brittle phases promotes microcrack initiation, and subsequent crack propagation may locally follow grain boundaries [17,35]. Overall, a large amount of eutectic structure and MC carbides are enriched near the crack, which is the fundamental reason why it is more prone to brittle fracture.

## 5. Conclusions

(1) The grain sizes of different modulus parts are controlled by the local cooling rates, and the cooling rates are related to moduli and the spatial position distributions. The tensile and yield strengths of the alloy are negatively correlated with the grain sizes. The cooling rate at the M_2.25_ area is the fastest, the grains are the finest, and the tensile and yield strengths are the highest. The cooling rate at the M_1.42_ section is the slowest, the grains are the coarsest, and the strength is the worst.
(2) The tensile and yield strengths of the alloy are negatively correlated with the sizes of the γ′ phases. A fine γ′ phase has a stronger dislocation hindrance capacity, resulting in greater tensile and yield strengths. The average size of γ′ phases at the M_2.25_ part is the smallest, which leads to its maximum strength. The average size of γ′ phases at the M_1.42_ part is the largest, which leads to its minimum strength.
(3) Elongation is related to the sizes of eutectic structure and MC carbides. Larger sizes of eutectic structure and MC carbides will intensify the tendency to fracture and reduce the plasticity of the alloy, and lead to a decrease in elongation. The elongation at the M_1.42_ part is the smallest and its plasticity is the worst, while the elongation at the M_1.00_ part is the largest and its plasticity is the best. When stretched at room temperature, cracks preferentially initiate at the eutectic structure and MC carbides in the vicinity of dendrites and grain boundaries.

## Figures and Tables

**Table 1:** Main composition of JG4246A alloy (wt.%).

Element	C	Cr	Mo	Ti	Al	Hf	W	Ni
composition	0.12	7.79	4.86	0.95	8.00	0.65	2.03	Bal.

**Table 2:** Maximum wall thicknesses and moduli of different feature sections.

Area	Maximum Wall Thickness/mm	Modulus M/mm
1#	20	2.25
2#	20.5	2.15
3#	9	1.65
4#	5	1.42
5#	2	1.00

## Data Availability

The original contributions presented in this study are included in the article. Further inquiries can be directed to the corresponding author.
